# Isolated Intracranial Recurrence of Burkitt Lymphoma Complicated by Dural Thickening: A Case Report

**DOI:** 10.7759/cureus.89791

**Published:** 2025-08-11

**Authors:** Yasuhiro Tanaka, Ryo Ikunari, Shusaku Take, Eiki Inoue

**Affiliations:** 1 Department of Hematology, Shinko Hospital, Kobe, JPN; 2 Department of Diagnostic Pathology, Kobe University Graduate School of Medicine, Kobe, JPN

**Keywords:** bl, cns recurrence, dural involvement, subdural hematoma, wbrt

## Abstract

Burkitt lymphoma (BL) usually involves extranodal regions such as the bone marrow and central nervous system (CNS), but dural involvement is rarely reported. We report a case of the intracranial recurrence of BL involving the CNS, leptomeninges, and dura mater. The patient was a 76-year-old woman who presented with systemic lymphadenopathies, bilateral pleural effusion, and ascites. Laboratory examination showed elevated white blood counts with circulating abnormal cells and thrombocytopenia. Bone marrow examination revealed the presence of abnormal medium-sized lymphocytes with cytoplasmic vacuoles, and IGH::MYC fusion gene was detected in these abnormal lymphocytes. Thus, the diagnosis of BL was made. No CNS lesions were identified at diagnosis. A complete metabolic response was achieved by rituximab combination chemotherapy with prophylactic intrathecal chemotherapy, but isolated intracranial recurrence of BL occurred. In intracranial recurrence, dural involvement was observed in addition to the leptomeninges. High-dose chemotherapy was not effective for relapsed BL, and she died because of disease progression. At autopsy, subdural hematoma (SDH) and dural thickening were pathologically found. This is a rare case of isolated intracranial BL recurrence with dural involvement and nontraumatic SDH as a complication, and we reviewed previous reports of secondary dural involvement in BL. Our case showed the important viewpoint that in the case of dural involvement, clinicians should be aware of the nontraumatic SDH as a complication, and whole-brain radiotherapy may be the optimal treatment for isolated intracranial BL recurrence with dural involvement after rituximab combination chemotherapy.

## Introduction

Burkitt lymphoma (BL) is a rare type of mature aggressive B-cell lymphoma and presents as a rapidly growing disseminated tumor. BL usually involves extranodal regions such as the bone marrow in addition to lymph nodes. BL cells show the characteristic translocation involving the *MYC* gene [1]. The standard therapy for BL is rituximab combination chemotherapy. BL recurs in nodal and extranodal sites such as bone marrow, large intestine, and breast; however, BL recurrence in the central nervous system (CNS) frequently occurs despite prophylactic intrathecal chemotherapy [2].

The standard treatment of BL recurrence in the CNS is CNS-directed chemotherapy such as high-dose methotrexate or cytarabine, or whole-brain radiotherapy (WBRT). The lesions in BL recurring in the CNS are mainly found in the parenchyma and leptomeninges, and dural thickening is rarely observed. In the case of dural involvement in B-cell lymphoma, nontraumatic subdural hematoma (SDH) occasionally occurs as a complication [3].

Here, we report a case of a female patient with isolated intracranial recurrence of BL. 

## Case presentation

A 76-year-old female patient was admitted to our hospital in January 2024 because of abdominal distention and nausea for two weeks. She had undergone right hip arthroplasty several years ago. Laboratory examination showed that her white blood count was 24400/µL with 11% circulating abnormal cells, hemoglobin level was 11.6 g/dL, and platelet count was 8.5x10^4^/µL. The level of C-reactive protein was 2.01 mg/dL, that of lactate dehydrogenase (LDH) was 3677 IU/L, and that of soluble interleukin-2 receptor (sIL-2R) was 6078 U/ml (Table 1).

**Table 1:** Results of laboratory tests WBC, white blood counts; RBC, red blood counts; CRP, C-reactive protein; LDH, lactate dehydrogenase; BUN, blood urea nitrogen; sIL-2R, soluble interluekin-2 receptor

Parameters (unit)	Patient Values (At Diagnosis)	Patient Values (At Recurrence)	Reference Range
WBC (/µL)	24400	5700	3300 - 8600
abnormal cells (%)	11	0	0
RBC (X10^4^ /µL)	396	341	386 - 492
Hemoglobin (g/dL)	11.6	10.9	11.6 - 14.8
hematocrit (%)	36.4	34.7	35.1 - 44.4
Platelets (X10^4^ /µL)	8.5	19.3	15.8 - 34.8
CRP (mg/dL)	2.01	0.08	below 0.14
Albumin (g/dL)	2.9	4.2	4.1 - 5.1
LDH (U/L)	3677	321	124 - 222
Uric acid (mg/dL)	15.6	3.9	2.6 - 5.5
BUN (mg/dL)	38	18	8 - 20
Creatinine (mg/dL)	2.15	0.67	0.46 - 0.79
sIL-2R (U/mL)	6078	436	122-496

**Figure 1:**
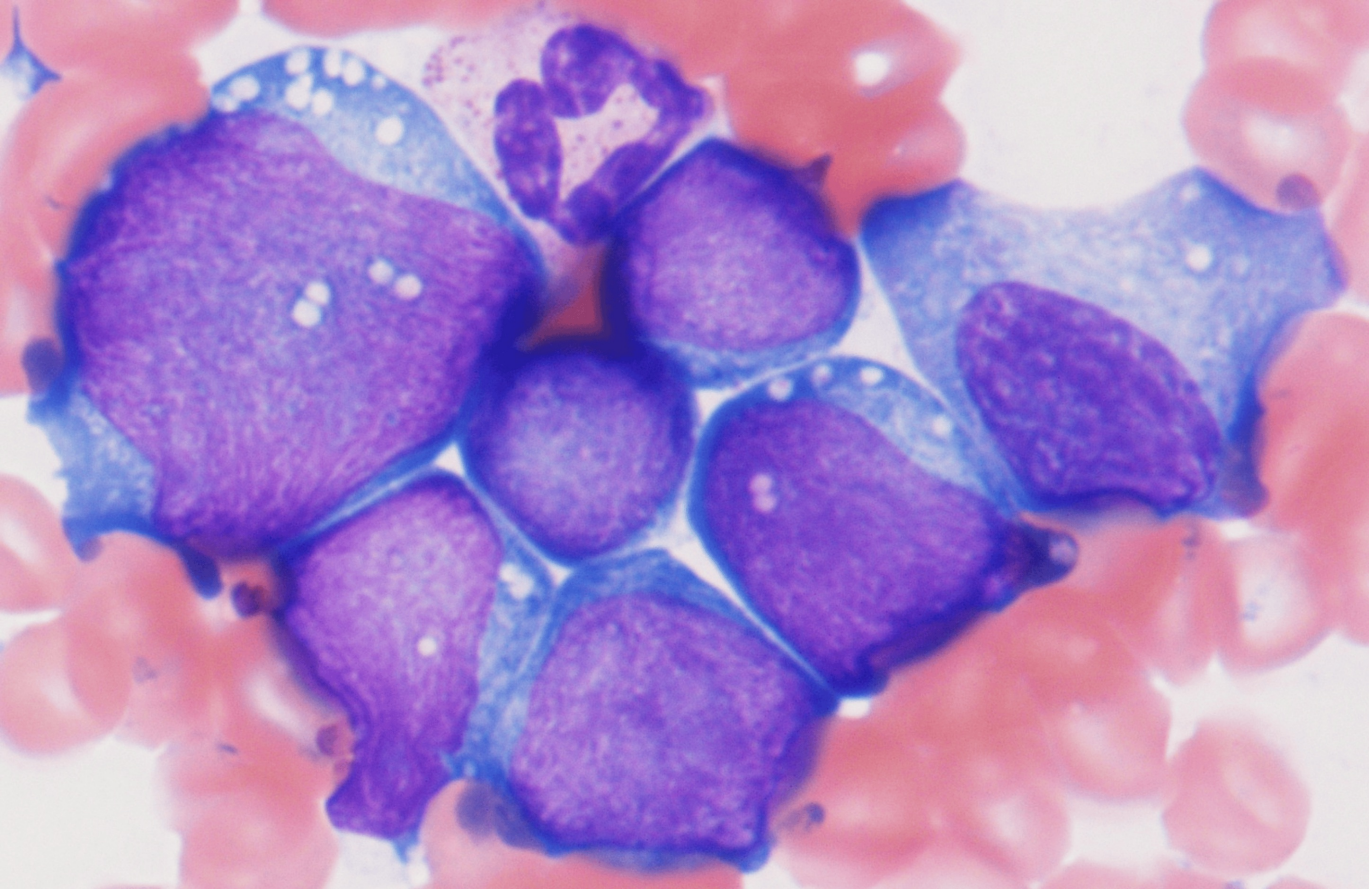
Smear specimen of bone marrow at diagnosis showing many medium-sized abnormal cells with cytoplasmic vacuoles (May-Giemsa, X 1000)

A serum test revealed that she was negative for human immunodeficiency virus-1 (HIV-1). Computed tomography (CT) showed systemic lymphadenopathies, bilateral pleural effusion, and ascites. Bone marrow examination showed many medium-sized abnormal cells with cytoplasmic vacuoles (Figure 1).

**Figure 2:**
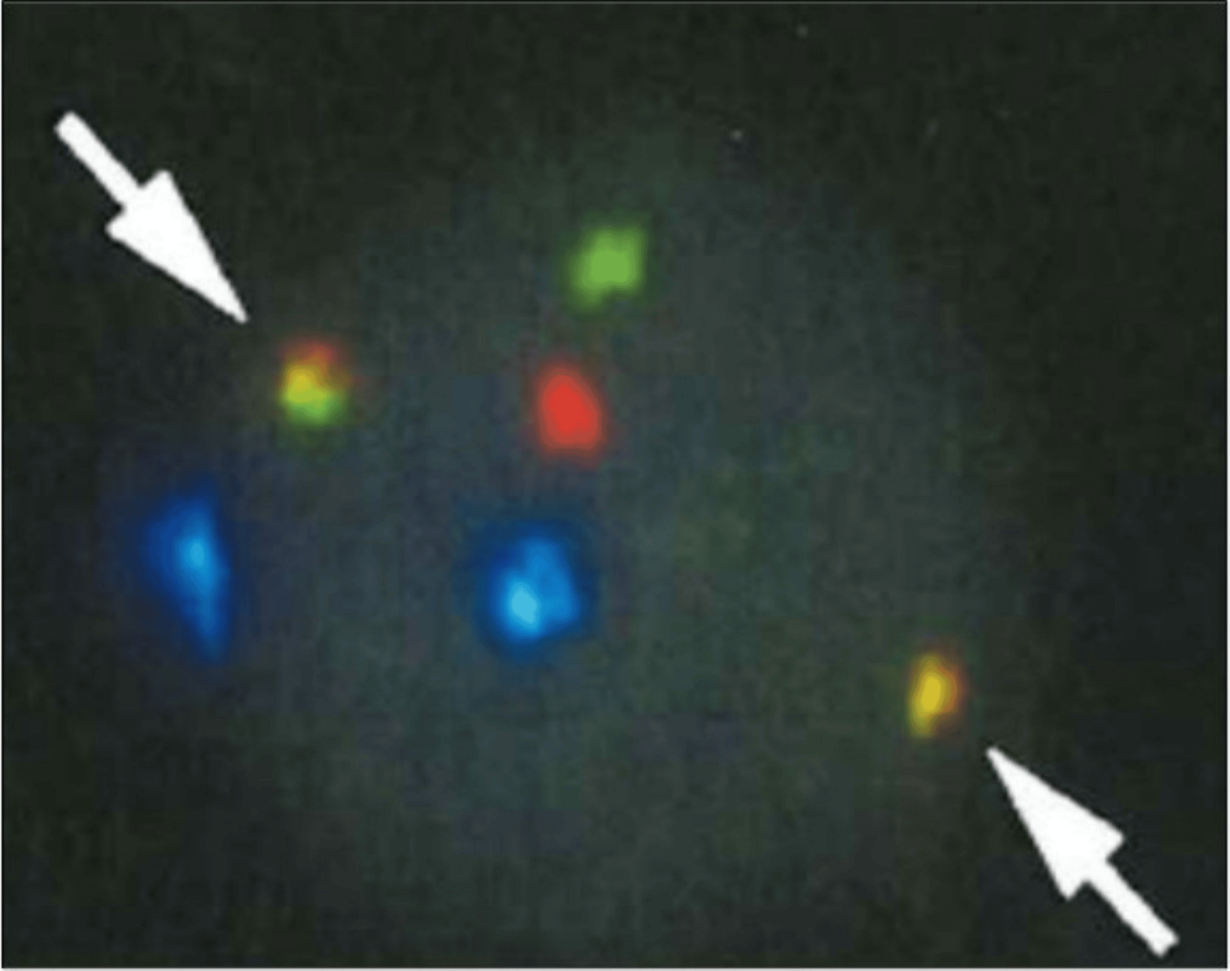
IGH::MYC fusion singals detected (white arrow) at diagnosis The yellow signal indicated the fusion signal, the green signal the IGH probe, the red signal the MYC probe, and the blue signal the D8Z2 probe

Flow cytometric analysis showed that these abnormal cells were positive for CD10, CD19, CD20, CD38, and kappa. Fluorescence in situ hybridization (FISH) analysis showed IGH::MYC fusion signals for these abnormal cells (55% of 100 analyzed cells) (Figure 2).

Epstein-Barr virus-encoded small RNAs (EBER) were negative for these abnormal cells by in situ hybridization. Chromosomal analysis using G banding showed 47, XX, add(1) (q32), t(8;14) (q24;q32), +18 (8/20 cells)/46, XX (12/20 cells). IgH rearrangement was detected by polymerase chain reaction (PCR). Plain brain magnetic resonance imaging (MRI) showed no abnormalities, and no abnormal cells were detected in the cerebrospinal fluid (CSF), suggesting that she did not have CNS lesions. Beta2-microglobulin and sIL-2R in the CSF were not measured. These results led to the diagnosis of HIV and EBV-negative BL, stage Ⅳ, in accordance with the World Health Organization (WHO) 2022 classification [1].

After the diagnosis, rituximab combination dose-adjusted EPOCH (DA-EPOCH-R) (etoposide, 60 mg per square meter vincristine, 0.4 mg per square meter and doxorubicin, 12 mg per square meter on days 1-4; cyclophosphamide, 900 mg per square meter on day 5; prednisolone, 60 mg per square meter on days 1-5; and rituximab, 375 mg per square meter on day 6) therapy starting at level 1 was administered [4]. After one course of DA-EPOCH-R therapy, positron emission tomography (PET)-CT images showed no abnormal accumulation of fluorodeoxyglucose, achieving a complete metabolic response (CMR). She received a total of six courses of DA-EPOCH-R therapy and eight courses of intrathecal injection of cytarabine, methotrexate, and prednisolone. She remained in remission until June 2024.

**Figure 3:**
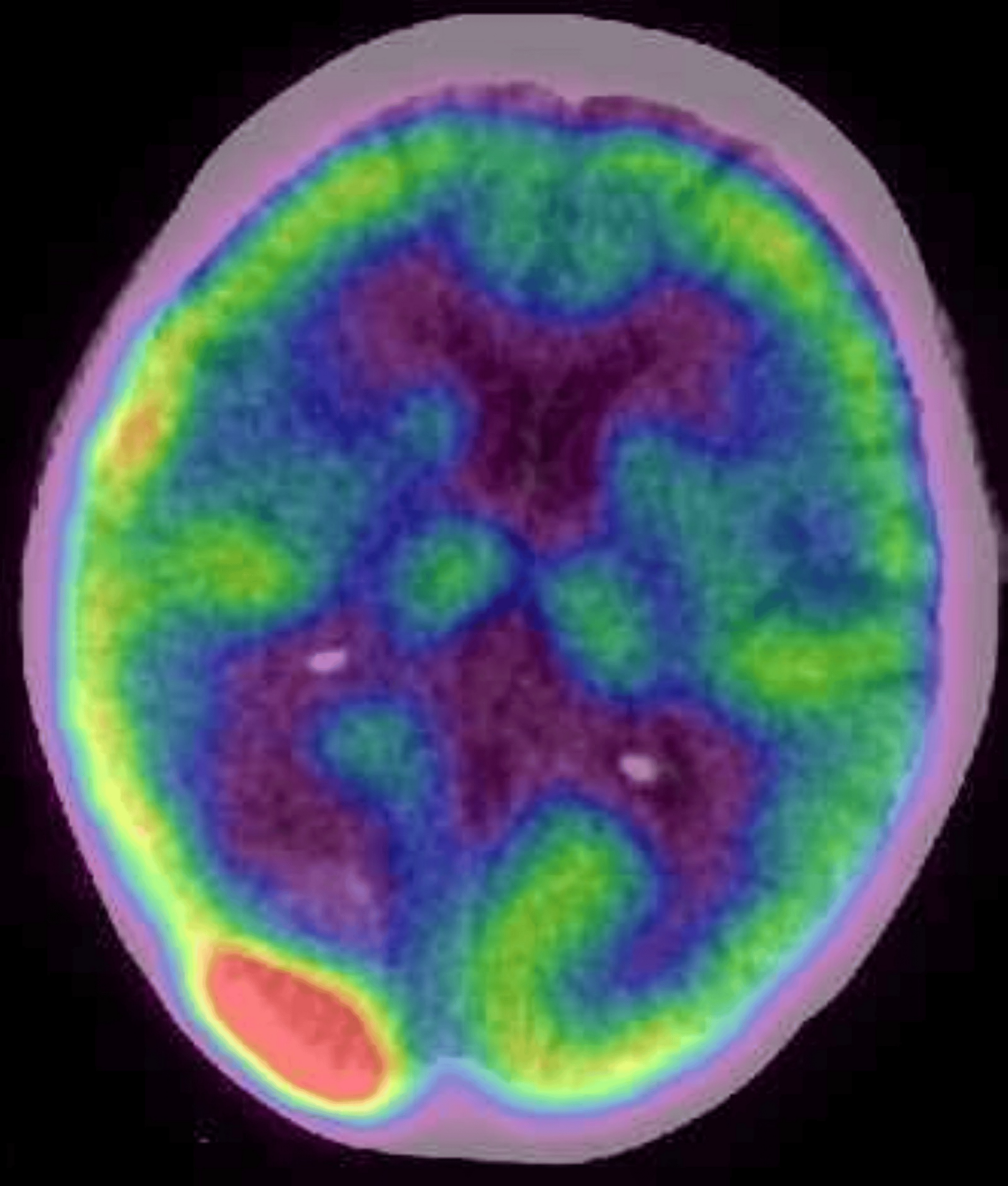
PET-CT image at relapse showing a mass with focal abnormal accumulation of fluorodeoxyglucose in the right occipital lobe PET, positron emission tomography; CT: computed tomography

However, she was readmitted to our hospital in July 2024 because of right visual disturbance and constriction of her visual field. PET-CT images taken one day before readmission revealed a mass with focal abnormal accumulation of fluorodeoxyglucose in the right occipital lobe (Figure 3).

**Figure 4:**
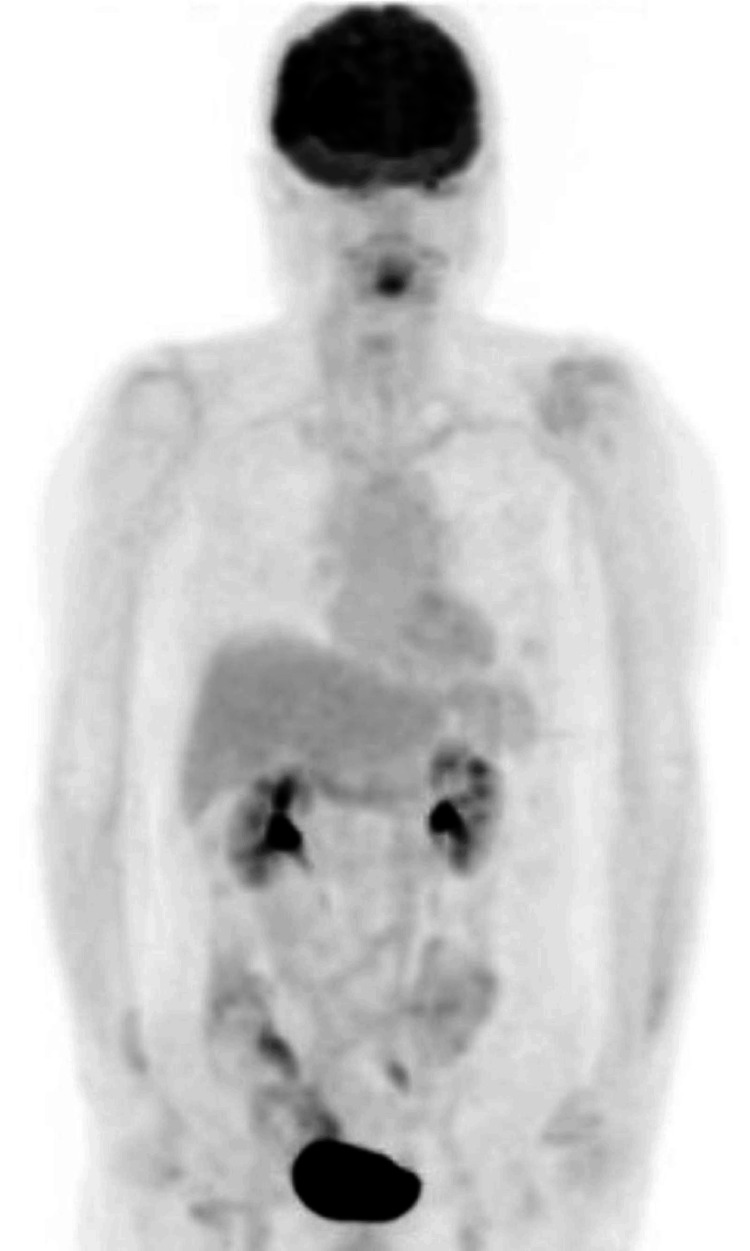
PET-CT image at relapse showing no abnormal accumulation of fluorodeoxyglucose in the thoracic and abdominal regions PET, positron emission tomography; CT: computed tomography

No abnormal accumulation of fluorodeoxyglucose was found in the thoracic and abdominal regions (Figure 4).

**Figure 5:**
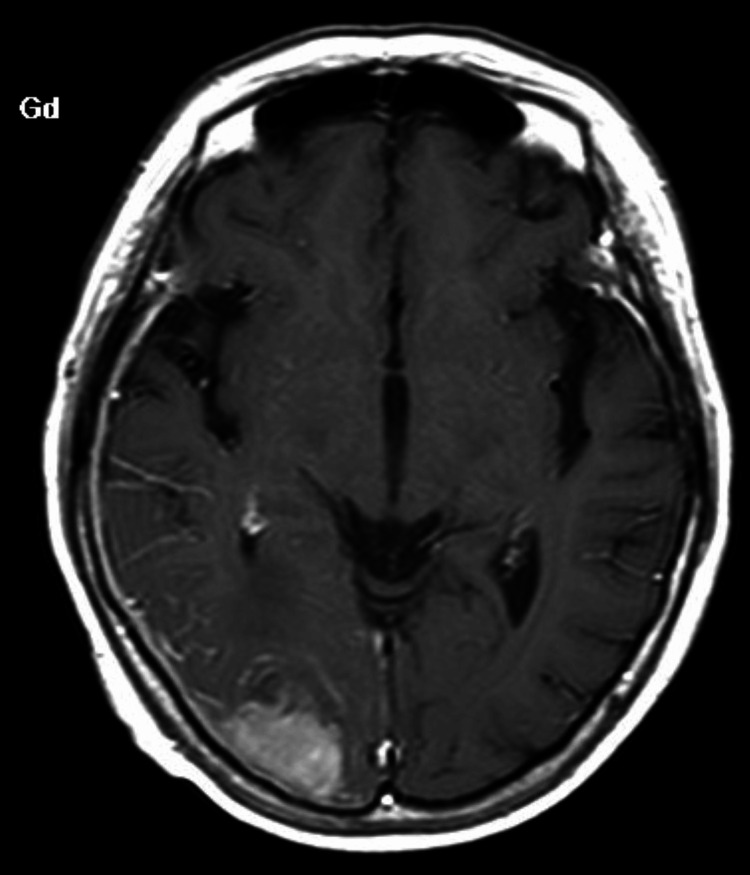
Contrast-enhanced brain MRI image at relapse reveals the mass showing heterogenous contrast enhancement; dural thickening is also observed from the right temporal area to the parietal area

Contrast-enhanced brain MRI showed a mass of about 3 cm in the right occipital lobe. The mass showed an iso-intensity on the T1-weighted image, a low-intensity on the T2-weighted image, and a heterogeneous contrast enhancement. Contrast-enhanced dural thickening was also found from the right temporal area to the parietal area (Figure 5).

**Figure 6:**
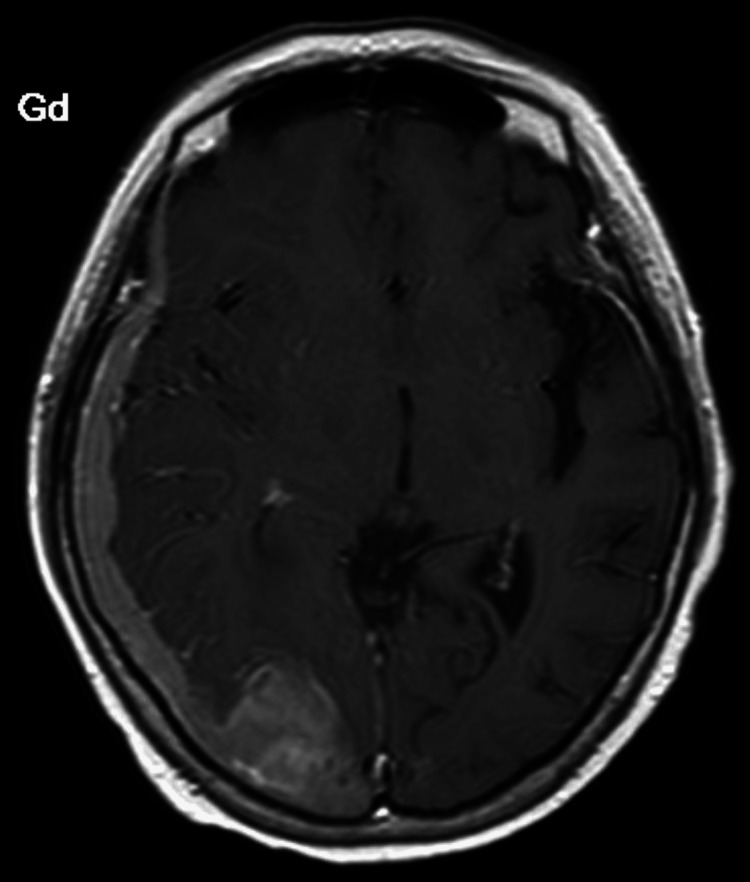
Contrast-enhanced brain MRI after chemotherapy showing enlargement of the mass with heterogenous contrast enhancement, and dural thickening worsened from the right temporal area to the parietal area

An open biopsy of the dura mater or parenchymal lesion was not performed. Many abnormal cells were detected in her CSF. Flow cytometric analysis showed that these abnormal cells were positive for CD10, CD19, CD38, and kappa, and negative for CD20. Chromosomal analysis and FISH analysis were not carried out. Laboratory examination showed no abnormalities except for the elevation of the LDH level to 321 IU/L (Table 1). No abnormal cells were found in the bone marrow. These results led to the diagnosis of isolated intracranial recurrence of BL involving the right occipital lobe, leptomeninges, and dura mater. High-dose methotrexate (3.5 g per square on day 1) and intermediate-dose cytarabine (1 g per square, twice a day, on days 1-2) were administered every two weeks. Her symptoms were transiently resolved, but loss of consciousness and left hemiparesis occurred after the chemotherapy. Contrast-enhanced brain MRI showed the enlargement of the mass in the right occipital lobe and greater thickening of the dura mater from the right temporal area to the parietal area (Figure 6).

**Figure 7:**
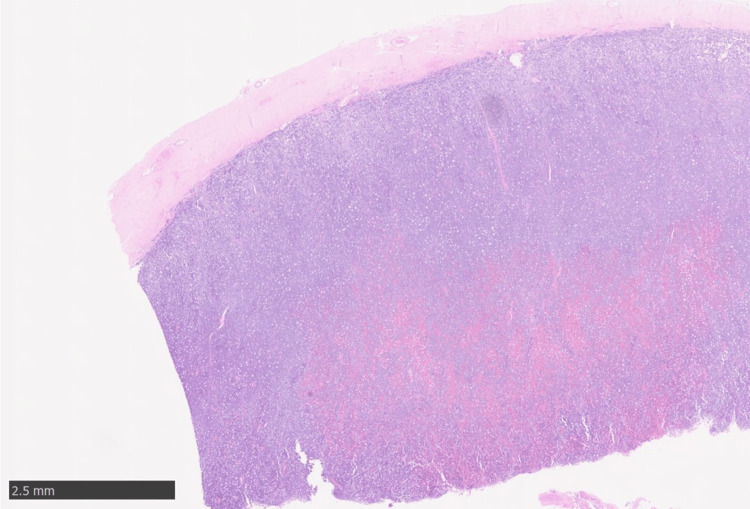
Brain autopsy findings showing thickened dura mater and hemorrhage and many abnormal cells in the subdural space (hematoxylin & eosin, X 1.25)

**Figure 8:**
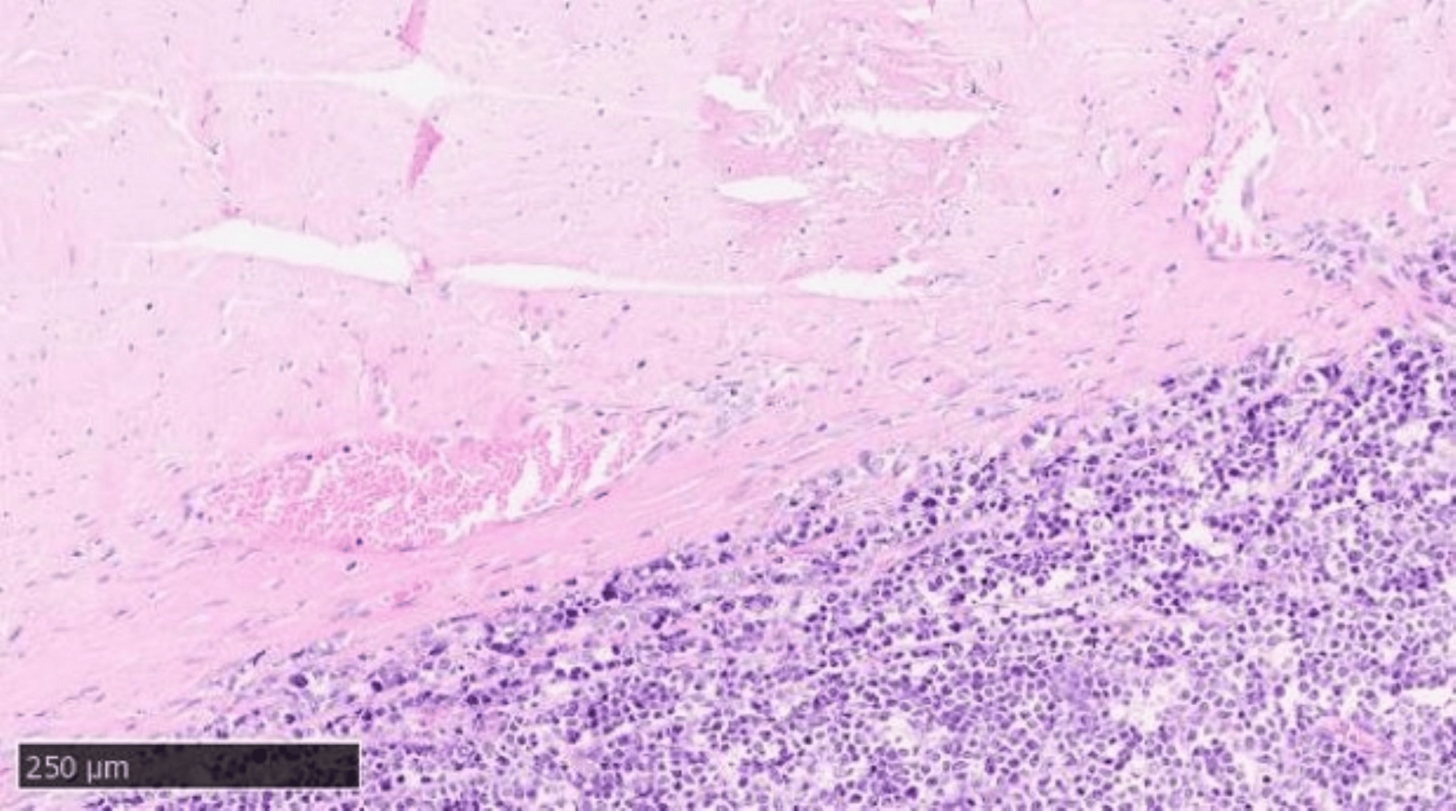
Brain autopsy findings showing many abnormal cells infiltrating from the subdural space into the dura mater (hematoxylin & eosin, X 400)

**Figure 9:**
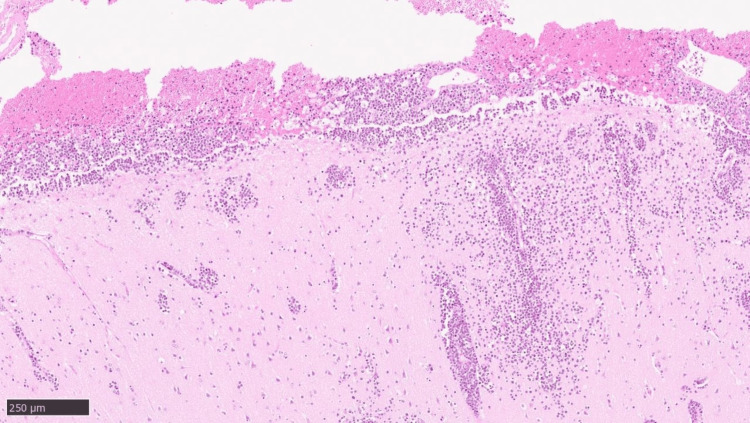
Brain autopsy findings showing many abnormal cells infiltrating from the subdural space into the brain parenchyma (hematoxylin & eosin, X 400)

Whole-brain radiotherapy (WBRT) was not performed because of her poor performance status (PS 4). The patient eventually died because of disease progression in August 2024. A brain autopsy was carried out with the permission of her family. On gross examination, the dura mater was tightly attached to the surface of the right temporal and occipital lobes, and many white nodules and hematomas were found on the surface of the brain under the dura mater. Microscopically, the dura mater was thickened, and hemorrhage and many abnormal cells were found in the subdural space (Figure 7). Many abnormal cells infiltrated from the subdural space into the dura mater (Figure 8) and the brain parenchyma (Figure 9).

**Figure 10:**
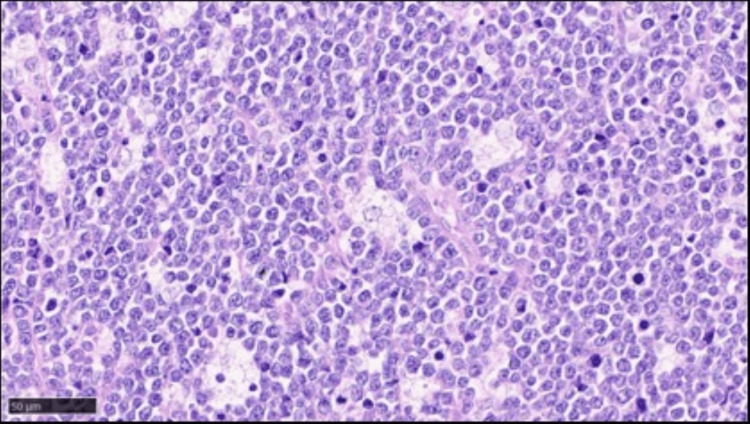
Brain autopsy findings showing proliferation of medium-sized abnormal cells and ocassional appearance of many histiocytes that phagocytosed apoptotic cells (starry-sky appearance) (hematoxylin & eosin, X 400)

These abnormal cells were medium-sized lymphoid cells, and many histiocytes that phagocytosed apoptotic cells (starry-sky appearance) were occasionally found (Figure 10).

**Figure 11:**
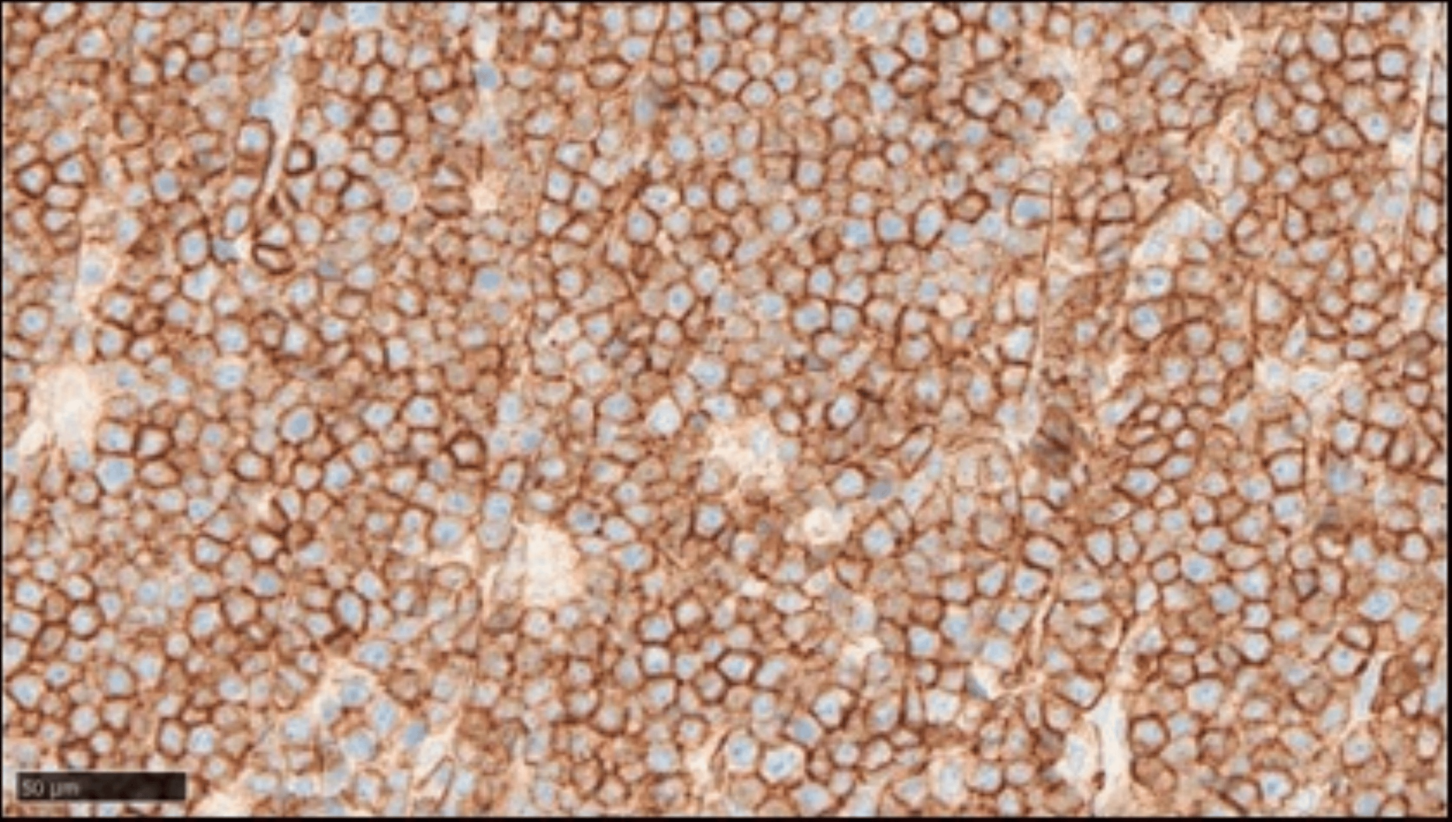
Immunohistochemical analysis (brain autopsy) showing that the abnormal cells are positive for CD20 (X 400)

**Figure 12:**
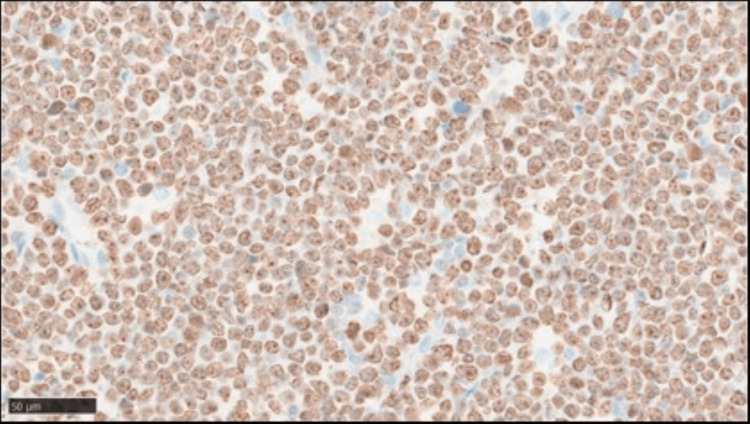
Brain autopsy findings showing Ki-67 labeling index is very high, above 95% (X 400)

Immunohistochemical analysis showed that these abnormal cells were positive for CD20 (Figure 11), and the Ki-67 labeling index was very high, above 95% (Figure 12).

This immunophenotyping was compatible with that in the bone marrow at the initial diagnosis. This confirmed the diagnosis of leptomeningeal BL recurrence involving the dura mater. From the autopsy findings, it was assumed that BL cells infiltrated into the extravascular space from the vessels of the subdural space and extended to the surface of the dura mater and the brain, and dural thickening and hemorrhage in the subdural space occurred.

## Discussion

Here, we reported a case of isolated intracranial recurrence of BL involving the right occipital lobe, leptomeninges, and dura mater. At autopsy, many BL cells and the hemorrhage occupied in the subdural space between the thickened dura and the parenchymal tissue of the brain.

The lesions in the CNS in BL are mainly in the parenchyma and leptomeninges, but dural thickening is rarely observed. Bahashwan et al. reviewed 36 cases of primary CNS BL, but dural involvement in primary CNS BL was not included in their review [5]. To the best of our knowledge, only three cases of secondary dural BL have been reported (Table 2) [6-8].

**Table 2:** Reported cases of secondary dural involvement in Burkitt lymphoma BM, bone marrow; LNs, lymph nodes; CSF, cerebrospinal fluid; SDH, subdural hematoma; WBRT, whole-brain radiotherapy; CR, complete remission; BL, Burkitt lymphoma

Authors, years	Age (years)/Sex	Onset in Dural Involvement	Involved Lesions	Intracranial Complication	Treatment	Outcome	Reference
Kitano et al., 2005	53/Male	Diagnosis	BM, CSF	SDH	Operation→systemic chemotherapy	Alive in CR	[6]
Wadhera et al., 2013	18/Male	Recurrence	Fontal lobe, CSF	Sagittal sinus occlusion	WBRT	Alive	[7]
Filho et al., 2016	21/Male	Diagnosis	BM, LNs, liver, spleen, pancreas	SDH	Operation→systemic chemotherapy	Death by sepsis	[8]
Tanala et al., 2025	76/Female	Recurrence	Occipital lobe, CSF	No	Systemic chemotherapy	Death by BL	Current Case

Of the three patients in the previously reported cases, one died of sepsis after systemic chemotherapy [8], and the others survived. Of the two survivors, one received the hyper-CVAD regimen with intrathecal injection of cytarabine or methotrexate for the leukemic phase of BL and achieved complete remission [6], and the second received WBRT for intracranial recurrence of BL after systemic chemotherapy [7].

Wadhera et al. reported a case of an 18-year-old man diagnosed as having BL involving the bone marrow and intestine [7]. The patient received systemic chemotherapy (RCOMP (rituximab, cyclophosphamide, non-pegylated liposomal doxorubicin, vincristine, and prednisone) regimen), but five months after the diagnosis, he complained of severe headaches and blurred vision. Brain MRI showed a focal parenchymal lesion in the right frontal lobe and dural-based lesions in the parieto-occipital region. Lymphomatous infiltration was detected in his CSF. The diagnosis of intracranial recurrence of BL was made. The patient received WBRT, and his symptoms resolved [7]. On the other hand, our patient relapsed six months after the initial diagnosis of BL, and brain MRI showed a mass of 3 cm in the right occipital lobe and dural thickening from the right temporal area to the parietal area. Our patient’s symptoms worsened despite systemic or CNS-directed chemotherapy, and she did not receive WBRT because of her poor PS. Therefore, WBRT may be the appropriate treatment strategy for BL with dural involvement after rituximab combination chemotherapy.

There are two main types of SDH: one is SDH related to predisposing factors such as trauma, and the other is spontaneous nontraumatic SDH [8]. Spontaneous nontraumatic SDH is mainly associated with hematological malignancies, with an incidence of 0.06-2.8% [9]. In the case of B-cell lymphoma involving the dura mater, spontaneous nontraumatic SDH occasionally occurred as a complication. Yagisawa et al. reported 14 cases of dural lymphoma complicated with nontraumatic SDH [3]. Most of them were low-grade lymphoma, such as mucosa-associated lymphoid tissue (MALT) lymphoma. Thus, nontraumatic SDH rarely occurs in aggressive lymphoma, including BL. Among the three previously reported cases of dural involvement in BL [6-8], two were complicated by SDH [6,8]. Of these, the first received the hyper-CVAD regimen after the burr-hole operation for SDH [6], and the second died of sepsis after the burr-hole operation and systemic chemotherapy [8]. However, the outcome of SDH in patients with hematological malignancies has not been determined by whether the operative management for SDH was performed or not.

Wright. et al. reported that in SDH in patients with hematological malignancies, no significant difference in 30-day or overall mortality was observed between the surgical group and the nonsurgical group, and the median survival of patients with the hematological malignancies complicated by SDH remains poor, that is, at less than five months of survival [9]. Thus, the systemic treatment strategy for hematological malignancies, not the choice of the operation for SDH, may determine the prognosis of patients with hematological malignancies complicated by SDH. One hypothesis of the mechanism of the development of SDH as a complication with dural metastasis is assumed to be as follows: cancer cells infiltrate into the tissue around the dura mater, thereby mechanically obstructing external dural vessels, leading to the dilatation and rupture of the capillaries of the inner dural layer [8]. The brain autopsy finding in the current case that BL cells infiltrated into the extravascular space from the vessels of the subdural space and extended to the surface of the dura mater and the brain may support this hypothesis.

## Conclusions

We reported a case of the intracranial recurrence of BL involving the right occipital lobe, leptomeninges, and dura mater following DA-EPOCH-R therapy and prophylactic intrathecal chemotherapy. At autopsy, many BL cells and the hemorrhage occupied the subdural space between the thickened dura and the parenchymal tissue of the brain. WBRT may be the optimal treatment strategy for dural involvement in BL recurrence after systemic chemotherapy. 
